# Inverse expression of ubiquitin-specific peptidase 19 and caspase 7 correlates with gastric neoplastic transformation

**DOI:** 10.1186/s13000-026-01777-9

**Published:** 2026-03-11

**Authors:** Philip Kelemen, Michael Naumann, Michael Vieth

**Affiliations:** 1 Institute of Pathology, Klinikum Bayreuth, Bayreuth, 95445 Germany; 2 Institute of Experimental Internal Medicine, Otto-von-Guericke-Universität Magdeburg, Magdeburg, 39120 Germany; 3 Friedrich-Alexander-Universität Erlangen-Nürnberg, Erlangen, 91054 Germany

**Keywords:** Deubiquitinylase, Gastric carcinogenesis, Gastric mucosa, HP-gastritis

## Abstract

**Background:**

Ubiquitin-specific protease 19 (USP19) is a deubiquitinylase (DUB) that is part of the USP family, the largest group of DUBs in humans. Growing evidence has indicated that USP19 is involved in tumor progression and serves as a new prognostic marker for various malignant disorders. Interestingly, USP19 has been shown to have both promoting and inhibiting effects on the onset and development of different neoplasms, depending on the specific tissue type. DUBs including USP19 affect a variety of cell functions including apoptotic cell death. Herein, caspase 7 acts as a key executioner in apoptosis, and its expression levels serve as a prognostic and diagnostic marker in various cancers. This study analysed the expression of USP19 and caspase 7 in gastric pathology along the progression of stomach cells to gastric adenocarcinoma (Correa cascade).

**Methods:**

We analysed the expression and subcellular localization of USP19 and caspase 7 by immunohistochemistry (IHC) in 296 paraffin-embedded human gastric tissue samples. The cohort included various gastric conditions such as autoimmune gastritis (A-gastritis), *Helicobacter pylori* gastritis (HP-gastritis), chemical gastropathy (C-gastritis), adenoma, and adenocarcinoma, using gastric mucosa without pathological changes as a reference point.

**Results:**

We observed a significant upregulation of USP19 expression in HP-gastritis, adenoma and adenocarcinoma. In contrast, caspase 7 was significantly upregulated in A-gastritis and HP-gastritis and significantly downregulated in both adenoma and adenocarcinoma.

**Conclusions:**

USP19 and caspase 7 showed distinct expression patterns across different gastric pathologies. Both USP19 and caspase 7 overexpression maybe associated with inflammation, while USP19 overexpression and no caspase 7 expression could indicate neoplastic transformation. This inverse expression may help distinguish early and late neoplastic epithelial changes in chronic gastritis.

**Supplementary Information:**

The online version contains supplementary material available at 10.1186/s13000-026-01777-9.

## Introduction

 Chronic gastritis is a long-standing inflammation of the gastric mucosa and represents a key step in the cascade toward gastric cancer [1]. Within the revised Sydney system, histological evaluation considers several parameters, including the degree of neutrophil activity, chronic inflammatory infiltrates, glandular atrophy, intestinal metaplasia, and the density of *Helicobacter pylori*. The infection with *H. pylori* is by far the most common cause (HP-gastritis), whereas autoimmune-mediated (A-gastritis) and chemically induced gastritis (C-gastritis) occur less frequently. As tissue damage advances, glands are lost and metaplastic changes appear which together create conditions favoring neoplastic development [2]. This sequence of chronic inflammation, atrophy, intestinal metaplasia, dysplasia, and carcinoma is known as the Correa cascade and represents the central pathway of *H. pylori*-associated gastric carcinogenesis [1]. Current international guidelines explicitly classify chronic atrophic gastritis and intestinal metaplasia as pre-cancerous conditions and recommend endoscopic surveillance in patients at increased gastric cancer risk [3].

The World Health Organization (WHO) classifies gastric adenomas as polypoid lesions formed by tubular and/or villous structures with dysplastic epithelium. High-grade adenomas show significant nuclear atypia and architectural disarray, while low-grade adenomas display mild to moderate atypia. These lesions occur most frequently in the gastric antrum and can develop sporadically or in association with familial adenomatous polyposis. Although histologically benign, some adenomas progress to adenocarcinoma within a short period, particularly when high-grade dysplasia is present [4, 5].

Gastric adenocarcinoma remains a major cause of cancer deaths, and its histology is remarkably variable. In clinical and research settings, the Lauren classification and the WHO classification are the most commonly used classification systems. Lauren differentiates intestinal, diffuse, and indeterminate types, whereas the WHO subdivides gastric adenocarcinomas into papillary, tubular, mucinous, and poorly cohesive forms, with the latter including signet ring cell carcinoma. To ensure that all gastric neoplasms can be consistently classified, the WHO framework also includes a wide range of uncommon histological variants [3].

Deubiquitinylases (DUBs) and their dysregulation are a linchpin for the development of gastric diseases [6]. DUBs regulate the ubiquitinylation of proteins and reverse the effects of E3 ubiquitin ligases. In this way, they influence protein localization, signaling activity, and protein stability. These regulatory functions tie them closely to critical pathways, including cell cycle regulation, DNA repair, programmed cell death, and immune system responses. Several cancer types, including gastric cancers, have been shown to exhibit DUB overexpression or mutation. As a result, DUBs are being considered more and more as potential targets for future therapeutic approaches as well as biomarkers [6].

In gastric cancer it has been shown that enhanced expression of the DUB USP19 correlated with cell proliferation and metastasis through reduced caspase 3 levels and increased MMP2/MMP9 expression [7]. USP19 has also been shown to inhibit cell proliferation in clear cell renal cell carcinoma, where its overexpression suppresses both proliferation and migration [8]. In addition, high USP19 expression has been reported as a favorable prognostic marker in high-grade serous ovarian carcinoma, suggesting a potential tumor-suppressive role in this context as well [9]. Blocking USP19 in a range of neoplasms, such as colorectal, breast, and stomach cancers as well as Ewing’s sarcoma, has potent anti-proliferative and anti-tumorigenic effects [10]. Overall, the role of USP19 as a cancer-relevant DUB with tissue-specific effects is recognized. Furthermore, it is important to analyse the changes in USP19 expression along different gastric pathologies immunohistochemically.

As mentioned above [6], DUBs including USP19 impact cell functions including apoptotic cell death. A main defense against malignant transformation is a tightly regulated program of cell death driven by caspases. Effector caspases are synthesized as inactive precursors and activated through cleavage by initiator caspases. After activation, they cleave essential cellular components, leading to apoptotic cell death. Caspases 3 and 7 act as executioner caspases, sharing some functions but also carrying out distinct tasks [11]. In gastric neoplasia a correlation of reduced caspase 3 level and increased USP19 expression was observed [7]. Although caspase 3 is related in structure and function to caspase 7, caspase 7 recognizes different substrates and plays an independent part in apoptosis. The presence of caspase 7 mutations and polymorphisms that impair its activity in several cancers indicates that caspase 7 may act as a tumor suppressor. Loss or reduction of caspase 7 activity could therefore compromise apoptotic capacity and enable tumor development [12]. Although the important role of caspase 7 as an executioner caspase is well known, there is no detailed immunohistochemical analysis of its expression in gastric pathology.

The current study determines USP19 expression levels in early stages of gastric disease, precursor lesions, and adenocarcinomas along the Correa cascade in gastric biopsies. In addition, we examined how caspase 7 expression occurs along the Correa cascade with the aim of identifying expression patterns that could be helpful in distinguishing between early stages of gastric disease and neoplastic epithelial changes.

## Methods

### Tissue sampling and data acquisition

A collection of gastric tissue samples obtained from the archives of the Institute of Pathology in Bayreuth, Germany was analysed. All specimens were fixed in 4% formalin and subsequently embedded in paraffin according to established protocols [13]. The analysed 296 paraffin-embedded samples comprised gastric mucosa with no pathological changes, as well as cases of A-gastritis, HP-gastritis, C-gastritis, adenomas, and adenocarcinomas.

All gastritis cases were endoscopic biopsies. In the adenoma group, 10 specimens were resections and 34 were biopsies. In the adenocarcinoma group, 3 specimens were resections and 40 were biopsies. The adenocarcinomas were taken from patients with previous and active *HP-*gastritis, only.

Each antrum and corpus site had two biopsies each and were taken from different patients since some diseases like chemical reactive gastritis or autoimmune gastritis are limited to antrum or corpus. Therefore autoimmune gastritis was shown as an extra analysis based on corpus biopsies, only. It depends on the gastritis status to what group the individuals were grouped to.

All gastritis cases were graded and diagnosed according to the updated Sydney system. *H. pylori* was tested clinically with rapid urease test and Warthin-Starry-Silver stain in histology.

Two pathologists in the pathology institute reviewed each section that had been stained with hematoxylin and eosin (HE) independently. The samples were chosen at random from routine clinical procedures done between 2012 and 2025 for either diagnosis or treatment.

### Immunohistochemistry

Using a microtome (Leica Mikrosysteme Vertrieb GmbH, Wetzlar, Germany), formalin-fixed, paraffin-embedded tissue slices (4 μm thick) were cut and prepared for immunohistochemical analysis. Immunohistochemistry was performed on whole tissue sections to evaluate the intensity and subcellular localization of protein expression. A section of each sample was stained with hematoxylin and eosin as part of standard diagnostic protocols, while other sections were used for the immunohistochemical detection of USP19 and cleaved caspase 7. After deparaffinization, staining was performed on the fully automated LEICA Bond III system (Leica Mikrosysteme Vertrieb GmbH, Wetzlar, Germany) using monoclonal antibodies against USP19 (ab93159, dilution 1:70) and cleaved caspase 7 (ab255818, dilution 1:1000), with antigen recovery based on EDTA for 20 min. The antibodies, obtained from Abcam (Discovery Drive, Cambridge Biomedical Campus, Cambridge, CB2 0AX, UK) were validated according to the manufacturer’s technical documentation. Human mammary tissue (for USP19) and colon tissue (for caspase 7) were utilised as positive controls based on the manufacturer’s recommendations. Negative controls were prepared by omitting the primary antibody to exclude nonspecific binding. Whole slide imaging was performed using the Hamamatsu NanoZoomer S360 scanner (Hamamatsu Photonics Deutschland GmbH, Herrsching, Germany), with a 20× objective lens (NA 0.75), achieving a scanning resolution of 0.23 μm/pixel.

### Evaluation of tissue samples

Immunohistochemically stained tissue sections were independently examined by two pathologists. All evaluations were carried out blindly with no information about the gastritis status and with no access to the H&E routine stain. The senior pathologist drew 10% random samples and compared the countings with 100% concordance.

Digital images were analysed using the NDP.view2 software (Hamamatsu Photonics Deutschland GmbH, Herrsching, Germany). We used the immunoreactive score (IRS) developed by Remmele and Stegner to measure immunoreactivity in all samples [14].

The IRS is a semiquantitative evaluation method that combines staining intensity and the number of positively stained cells into a 12-point composite scale. A 3-point scale was used to rate the intensity of the staining (1 = weak, 2 = moderate, 3 = strong), and a 4-point scale was used to rate the percentage of stained cells (1 = < 10%, 2 = 10–50%, 3 = 51–80%, 4 = > 80%) (see below).


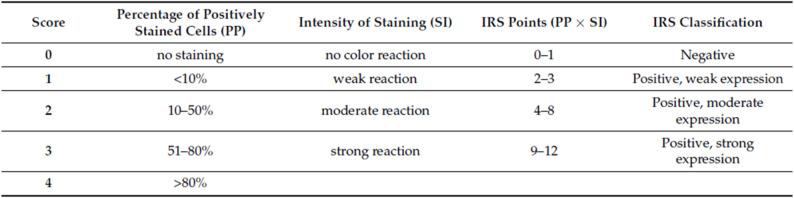


The final IRS was calculated by multiplying both scores, yielding a range of 1 to 12. Based on that, samples were divided into three groups: weakly positive (IRS 2–3), moderately positive (IRS 4–8), or strongly positive (IRS 9–12). There are no IRS values between 0 and 1. Therefore, we have only included the frequency for IRS values 2–3 (weak), 4–8 (moderate), and 9–12 (strong) in Tables 1 and 2. No average intensity was used. This scoring system gave us a standard way to measure and compare immunoreactivity between the groups. 

**Table 1:** Immunohistochemical staining intensity of cytoplasmic USP19 in gastric epithelium indicated by number of specimens in non-pathological and different pathological gastric tissues

Diagnosis	Number	M	Sex	F	Weak	Staining intensity Moderate	Strong
Normal	52	25		27	3 (5.8%)	35 (67.3%)	14 (26.9%)
Corpus	45				3 (6.7%)	32 (71.1%)	10 (22.2%)
Antrum	7					3 (42.9%)	4 (57.1%)
A-gastritis	52	17		35	2 (3.9%)	36 (69.2%)	14 (26.9%)
Corpus	52				2 (3.9%)	36 (69.2%)	14 (26.9%)/5*
*HP*-gastritis	53	31		22		25 (47.2%)	28 (52.8%)
Corpus	36					18 (50%)/2*	18 (50%)/6*
Antrum	17					7 (41.2%)	10 (58.8%)/5*
C-gastritis	52	38		14	5 (9.6%)	41 (78.9%)	6 (11.5%)
Antrum	52				5 (9.6%)	41 (78.9%)	6 (11.5%)/3*
Adenoma	44	27		17		12 (27.3%)	32 (72.7%)
Corpus	20					6 (30%)	14 (70%)
Antrum	24					6 (25%)	18 (75%)
Adenocarcinoma	43	19		24		18 (41.9%)	25 (58.1%)
Corpus	22					7 (31.8%)	15 (68.2%)
Antrum	18					11 (61.1%)	7 (38.9%)
Gastric remnant	3						3 (100%)

*Cases with intestinal metaplasia

**Table 2:** Immunohistochemical staining intensity of nuclear caspase 7 in gastric epithelium indicated by number of specimens in non-pathological and different pathological gastric tissues

Diagnosis	Number	M	Sex	F	Weak	Staining intensity Moderate	Strong
Normal	48	21		27	6 (12.5%)	38 (79.2%)	4 (8.3%)
Corpus	41				6 (14.6%)	32 (78.1%)	3 (7.3%)
Antrum	7					6 (85.7%)	1 (14.3%)
A-gastritis	50	16		34	1 (2%)	28 (56%)	21 (42%)
Corpus	50				1 (2%)	28 (56%)/3*	21 (42%)/1*
*HP*-gastritis	53	31		22		32 (60.4%)	21 (39.6%)
Corpus	36					19 (52.8%)/2*	17 (47.2%)/7*
Antrum	17					13 (76.5%)/1*	4 (23.5%)/3*
C-gastritis	52	38		14	1 (1.9%)	38 (73.1%)	13 (25%)
Antrum	52				1 (1.9%)	38 (73.1%)	13 (25%)/3*
Adenoma	44	29		15	21 (47.7%)	19 (43.2%)	4 (9.1%)
Corpus	20				9 (45%)	10 (50%)	1 (5%)
Antrum	24				12 (50%)	9 (37.5%)	3 (12.5%)
Adenocarcinoma	42	19		23	13 (31%)	29 (69%)	
Corpus	22				6 (27.3%)	16 (72.7%)	
Antrum	17				6 (35.3%)	11 (64.7%)	
Gastric remnant	3				1 (33.3%)	2 (66.7%)	

*Cases with intestinal metaplasia

Tables 1 and 2 report observed counts and percentages only. No statistical tests were applied to the tabulated data. Values are given as percentage. Where applicable, the number after the slash indicates the subset of cases with intestinal metaplasia within that category. Intestinal metaplasia was defined by the presence of goblet cells in the assessment area and the number indicates the subset of cases within that category [15].

Glandular atrophy and intestinal metaplasia were graded according to the updated Sydney system, and OLGA/OLGIM stages were assigned following current recommendations. The distribution of OLGA and OLGIM stages by gastritis etiology (A‑gastritis and HP‑gastritis) are summarized in Supplementary Tables S1 and S2, documenting the range of atrophy and metaplasia severity in our cohort.

### Statistical analysis

All independent, nonparametric data were processed in RStudio (v 2025.09.2 + 418, R Foundation for Statistical Computing). Each group (A-gastritis, HP-gastritis, C-gastritis, adenoma, and adenocarcinoma) was compared directly with non-pathological tissue, resulting in five comparisons per marker (Table S3). For each marker, the cytoplasmic and nuclear staining were assessed beforehand. For USP19, only the cytoplasmic IRS was analysed, as the nuclear IRS was difficult to assess. For caspase 7, only nuclear IRS was analysed because cytoplasmic IRS differences were less pronounced. For testing, antrum and corpus samples were pooled within each diagnostic category.

Group differences were assessed with two-sided Wilcoxon rank-sum tests (Mann–Whitney U) for independent samples (R function wilcox.test). To account for multiple comparisons, p-values were adjusted within each marker using the Benjamini–Hochberg procedure to control the false discovery rate, and only BH-adjusted p-values are reported and interpreted [16].

In addition to adjusted p-values, we report an effect estimate for each comparison using the two-sample Hodges–Lehmann shift (reported as normal minus disease) with a 95% confidence interval obtained by nonparametric bootstrap resampling within each group (5,000 resamples; percentile CI). Using thousands of bootstrap replications is standard practice for confidence limits, and the bootstrap literature explicitly discusses replication counts in the range of a few thousands (e.g., B ≈ 2000) and how larger B reduces Monte Carlo error in the interval endpoints [17].

Additionally, the corresponding effect estimates (Hodges-Lehmann shifts) with 95% confidence intervals and BH-adjusted p-values for all comparisons versus non-pathological mucosa are shown in Table S3.

A P value less than 0.05 was considered statistically significant. In all figures, adjusted P values are encoded as follows: A single asterisk (*) marks a statistically significant difference (p *<* 0.05), whereas a double asterisk (**) indicates a highly significant difference (p *<* 0.01).

## Results

### Overexpression of USP19 in HP-gastritis and neoplastic lesions

**Fig. 1:**
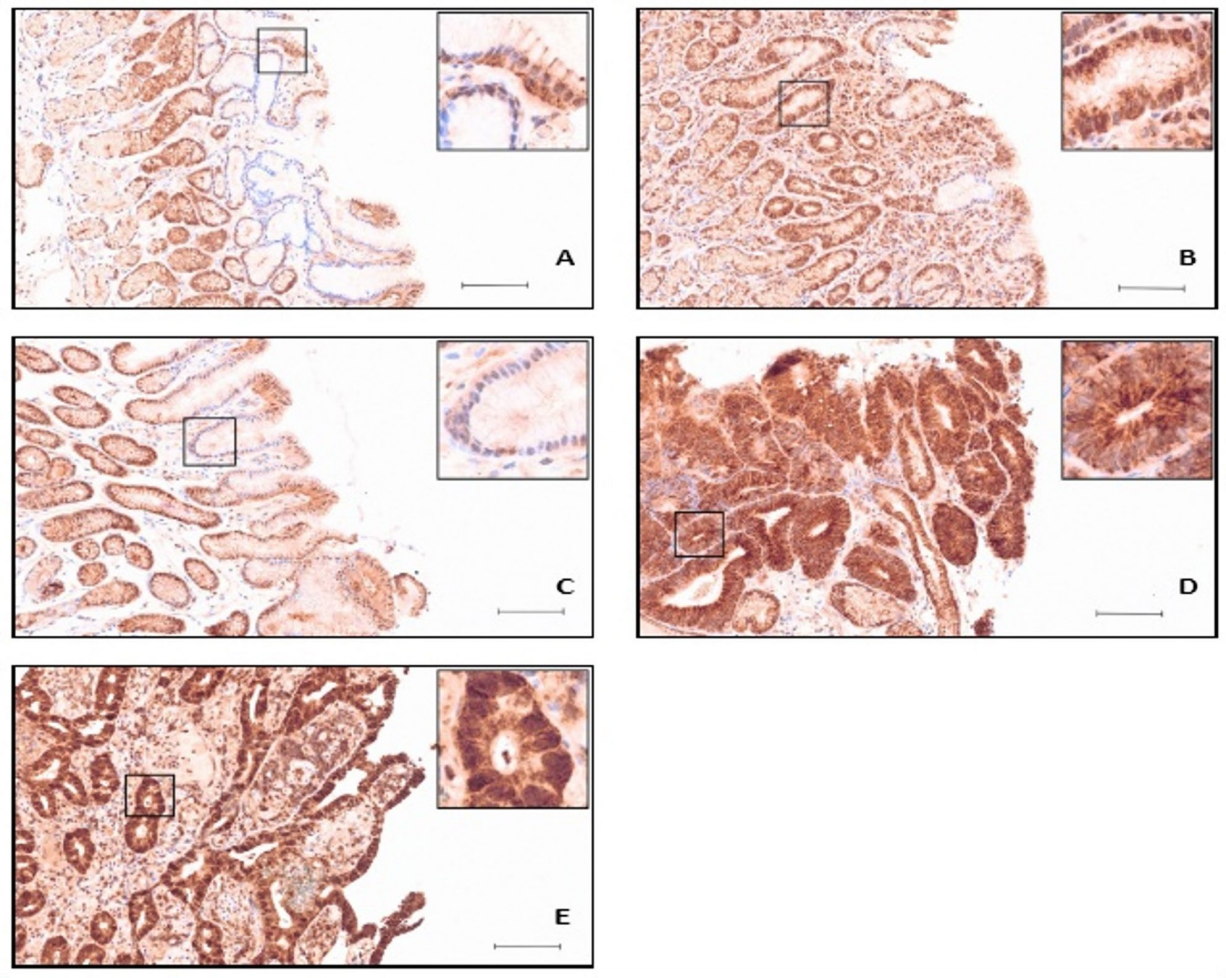
Immunohistochemical staining of USP19 in non-pathological and pathological gastric antrum tissue. Paraffin-embedded tissue sections were stained as described in methods. Representative images and magnifications are shown: **A** Moderate staining intensity in non-pathological gastric epithelium. **B** Stronger staining in HP-gastritis. **C** Weak staining intensity in C-gastritis. **D** Strong staining intensity in adenoma. **E** More prominently in adenocarcinoma. Scale bar: 100 µm

**Fig. 2:**
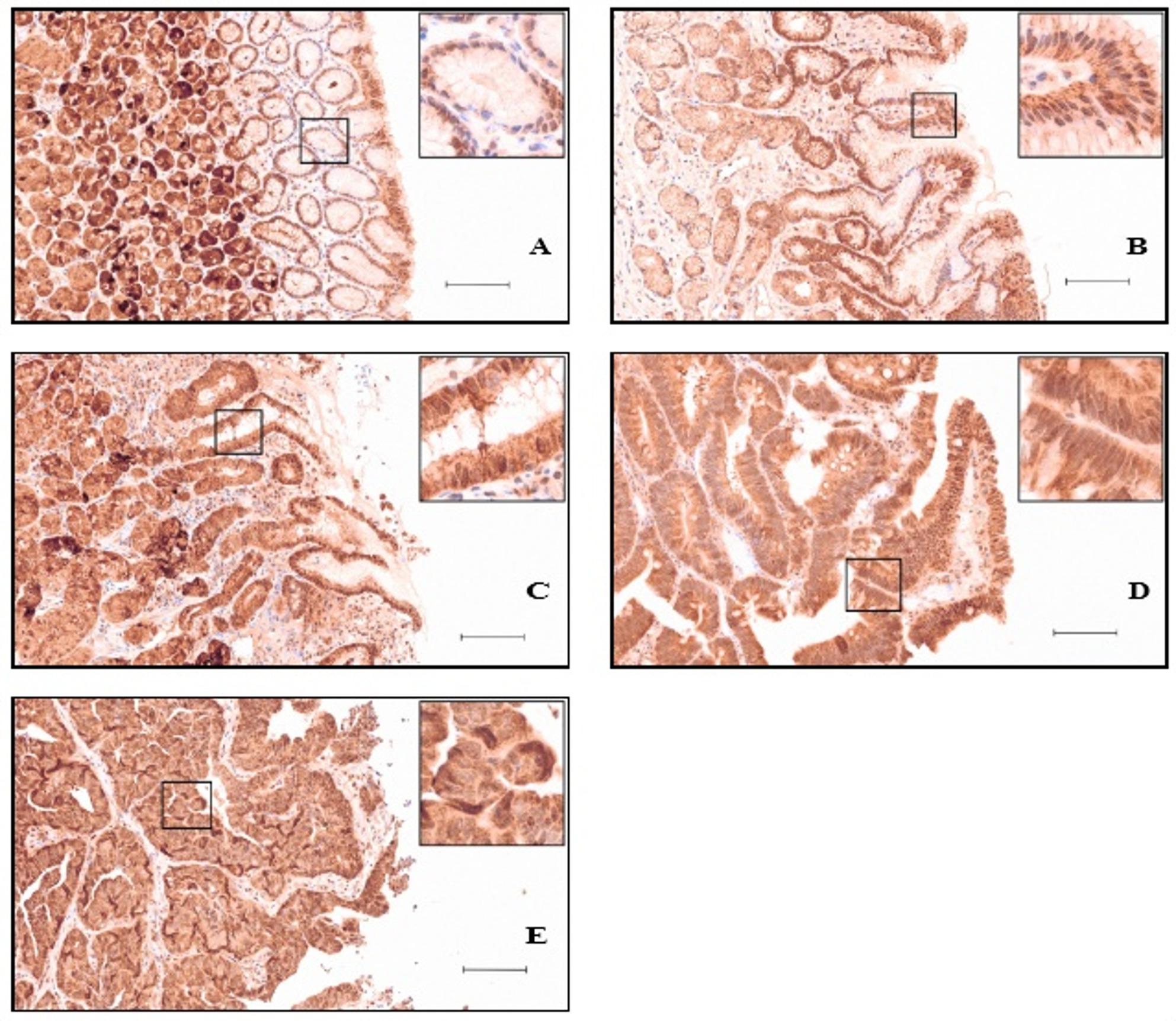
Immunohistochemical staining of USP19 in non-pathological and pathological gastric corpus tissue. Paraffin-embedded tissue sections were stained as described in methods. Representative images and magnifications are shown: **A** Moderate staining intensity in non-pathological gastric epithelium, **B** as well as in A-gastritis. **C** Stronger staining intensity in HP-gastritis. **D** Strong and diffuse staining intensity in adenoma and **E** more prominently in adenocarcinoma. Scale bar: 100 µm

Immunohistochemical staining of cytoplasmic USP19 expression was analysed across non-pathological and pathological gastric tissue. Scattered lymphocytes and plasma cells in the lamina propria showed weak USP19 positivity. No significant variation in staining intensity was observed between the surface foveolar and deep glandular epithelium, nor between the gastric antrum and corpus (Figs. 1 and 2). However, some unspecific parietal cell staining for unknown reason was observed. Immunohistochemical staining intensity of cytoplasmic USP19 in gastric epithelium was indicated by number of specimens and percentage in non-pathological and different pathological gastric tissues (Table 1).

In non-pathological tissue, USP19 staining most commonly fell into the moderate IRS category (IRS 4–8), which we regard as the baseline range of epithelial USP19 expression in this cohort. We consider strong IRS scores (IRS 9–12) as overexpression. When we sorted the non-pathological samples by location, the proportion of strong cases appeared to be higher in the antrum. However, this should be interpreted cautiously because the normal antrum subgroup was small (*n* = 7). Thus, this reflects variation rather than a consistent site-specific baseline difference.

**Fig. 3:**
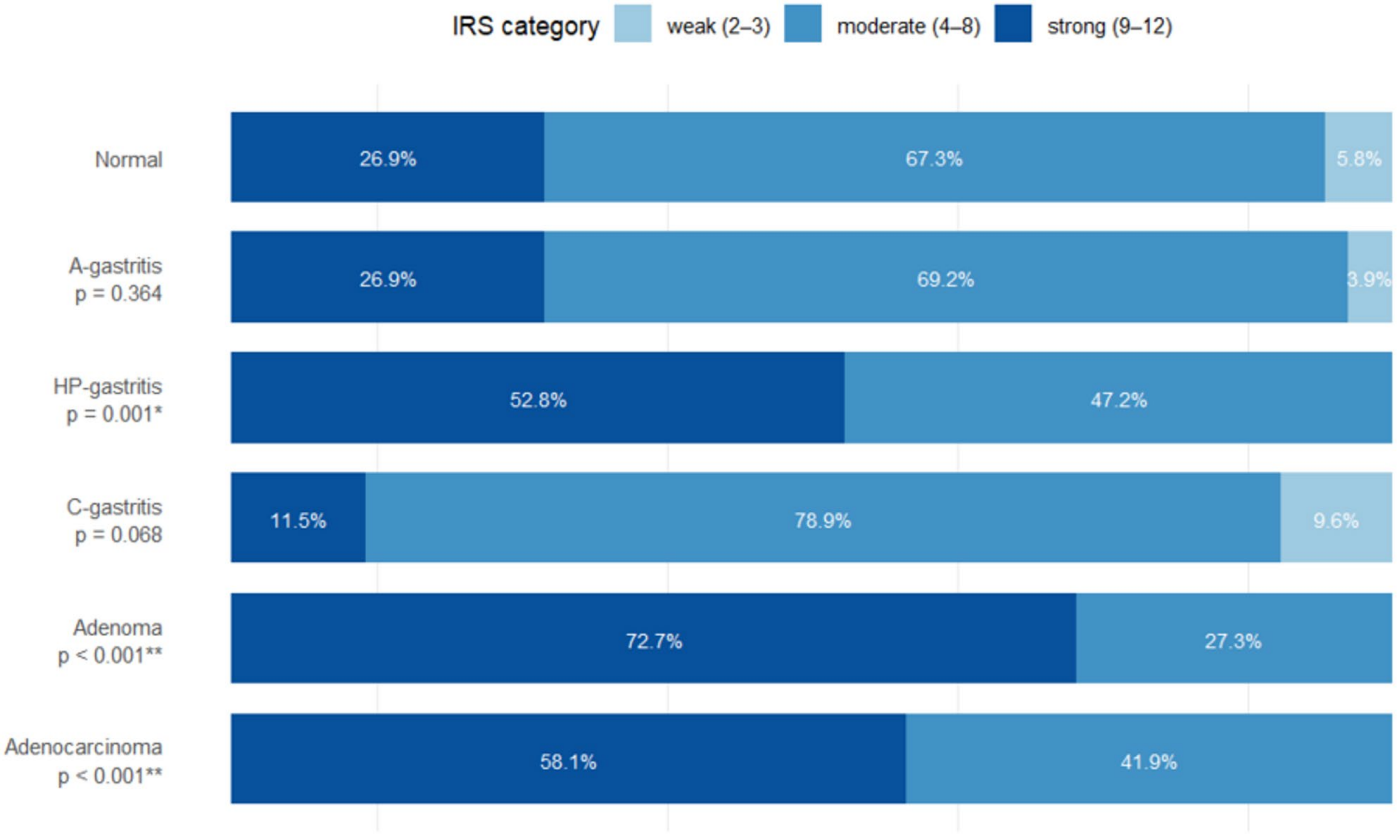
IRS of cytoplasmic USP19 expression in different gastric pathologies. Comparison of the percentage of cases across different gastric pathologies (including p-values). Each horizontal bar represents all cases within one entity. Bars are segmented according to staining intensity. Percentages shown inside the bar indicate the fraction of specimens in the corresponding category (values <3 % are omitted for legibility)

Cytoplasmic USP19 expression across diagnostic groups is represented by the proportions of cases in the predefined IRS categories (weak, moderate, strong) (Fig. 3). In non-pathological mucosa and A-gastritis, staining of USP19 is predominantly moderate (normal 67,3%, A-gastritis 69,2%) with a smaller strong fraction (normal 26,9%, A-gastritis 26,9%) (Fig. 3). The underlying case-level IRS distributions and the corresponding statistical comparisons versus non-pathological mucosa indicate that in A-gastritis, USP19 expression closely resembled normal mucosa (*p* = 0.364, Fig. 3).

In contrast, HP-gastritis showed a clear shift toward higher USP19 expression with more than half of cases classified as strong (52,8%), and neoplastic lesions show the most pronounced expression (adenoma 72,7%, and adenocarcinoma 58,1%). Adenocarcinoma cases are sporadic (the subgroups would be too small for tumor grades, which would lead to insufficient statistical significance). C-gastritis displays an opposite tendency with comparatively fewer strong cases (12%) (Fig. 3). Figure 3 shows all the underlying case-level IRS distributions and the corresponding statistical comparisons versus non-pathological mucosa.

### Caspase 7 increases in HP-and A-gastritis, but declines in neoplastic lesions

**Fig. 4:**
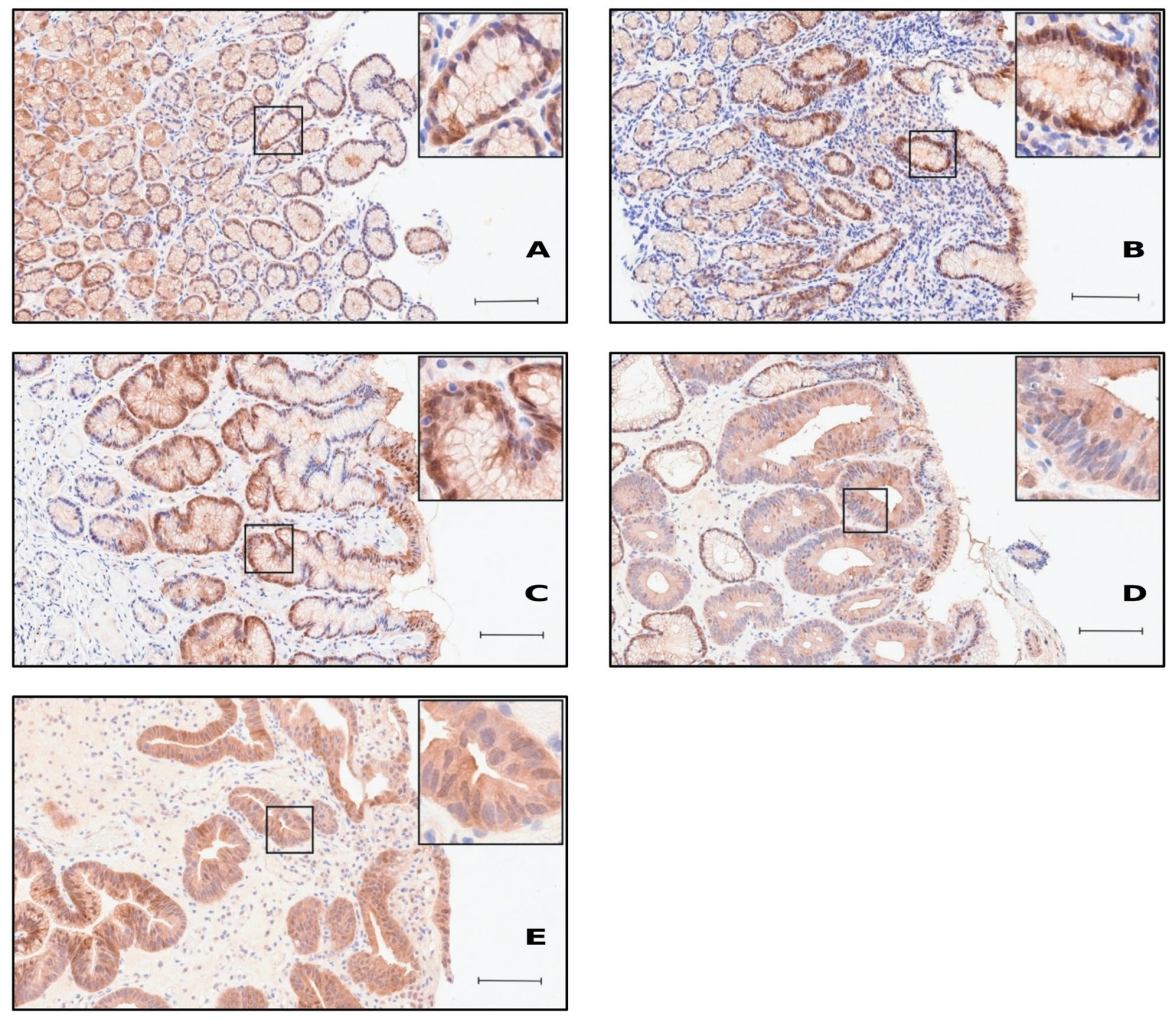
Immunohistochemical staining of caspase 7 in non-pathological and pathological gastric antrum tissue. **A** Moderate staining intensity in non-pathological gastric epithelium, **B** and stronger staining in inflammation in HP-gastritis. **C** Moderate staining intensity in C-gastritis. **D** Weak staining intensity in adenoma and **E** adenocarcinoma. Scale bar: 100 µm

**Fig. 5:**
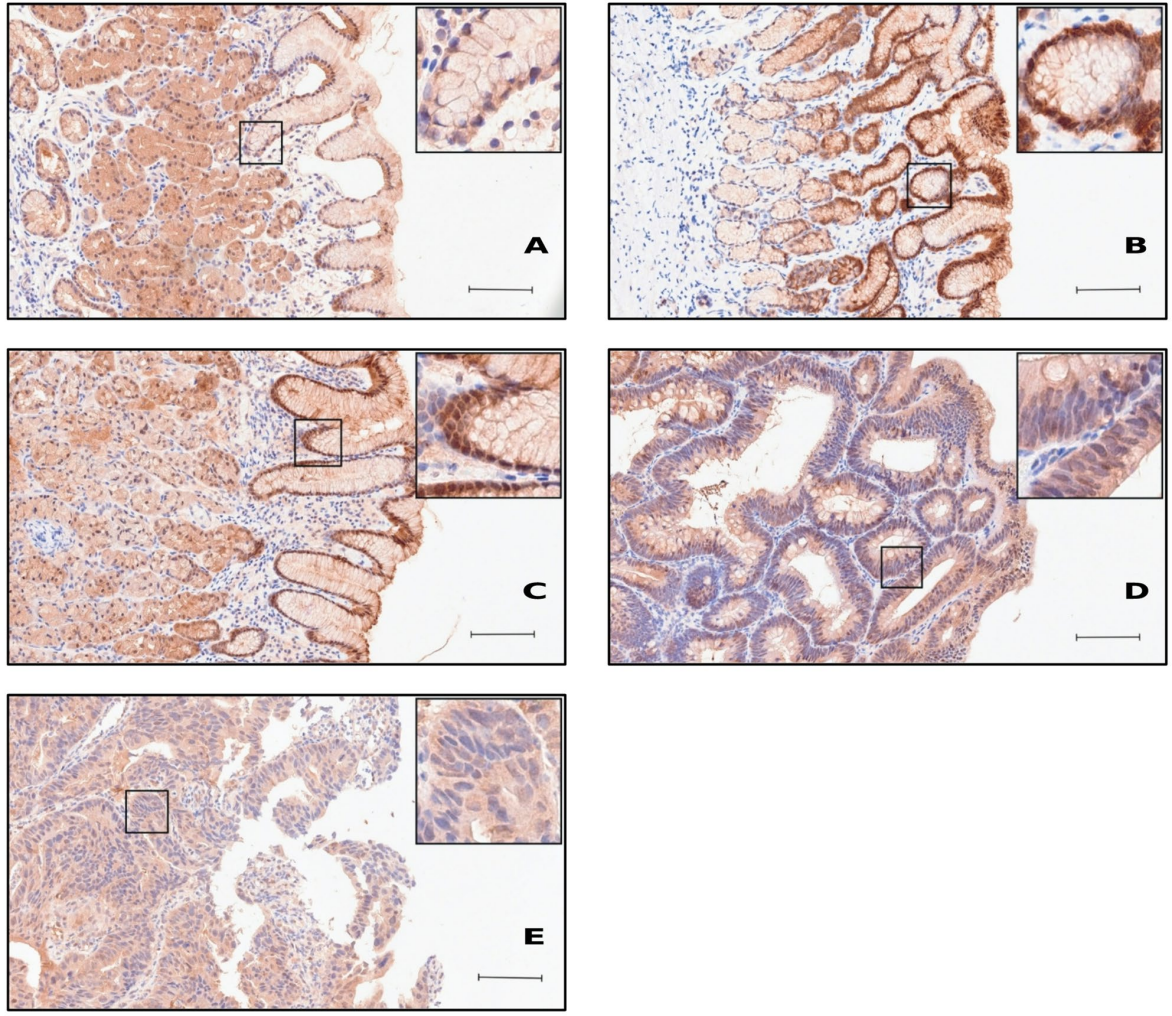
Immunohistochemical staining of caspase 7 in non-pathological and pathological gastric corpus tissue. **A** Moderate to strong staining intensity in non-pathological gastric epithelium, **B** as well as in A-gastritis. **C** Moderate to strong staining intensity in HP-gastritis. **D** Weak staining intensity in adenoma and **E** adenocarcinoma. Scale bar: 100 μm

Nuclear caspase 7 staining showed a disease-stage profile distinct from USP19. Cytoplasmic caspase 7 staining was also observed, but showed less pronounced differences and was therefore not included in the analysis. Similar to USP19, scattered lymphocytes and plasma cells showed weak positivity (Figs. 4 and 5). Nuclear immunohistochemical staining intensity of caspase 7 in gastric epithelium was indicated by number of specimens and percentage in non-pathological and different pathological gastric tissues (Table 2).

**Fig. 6:**
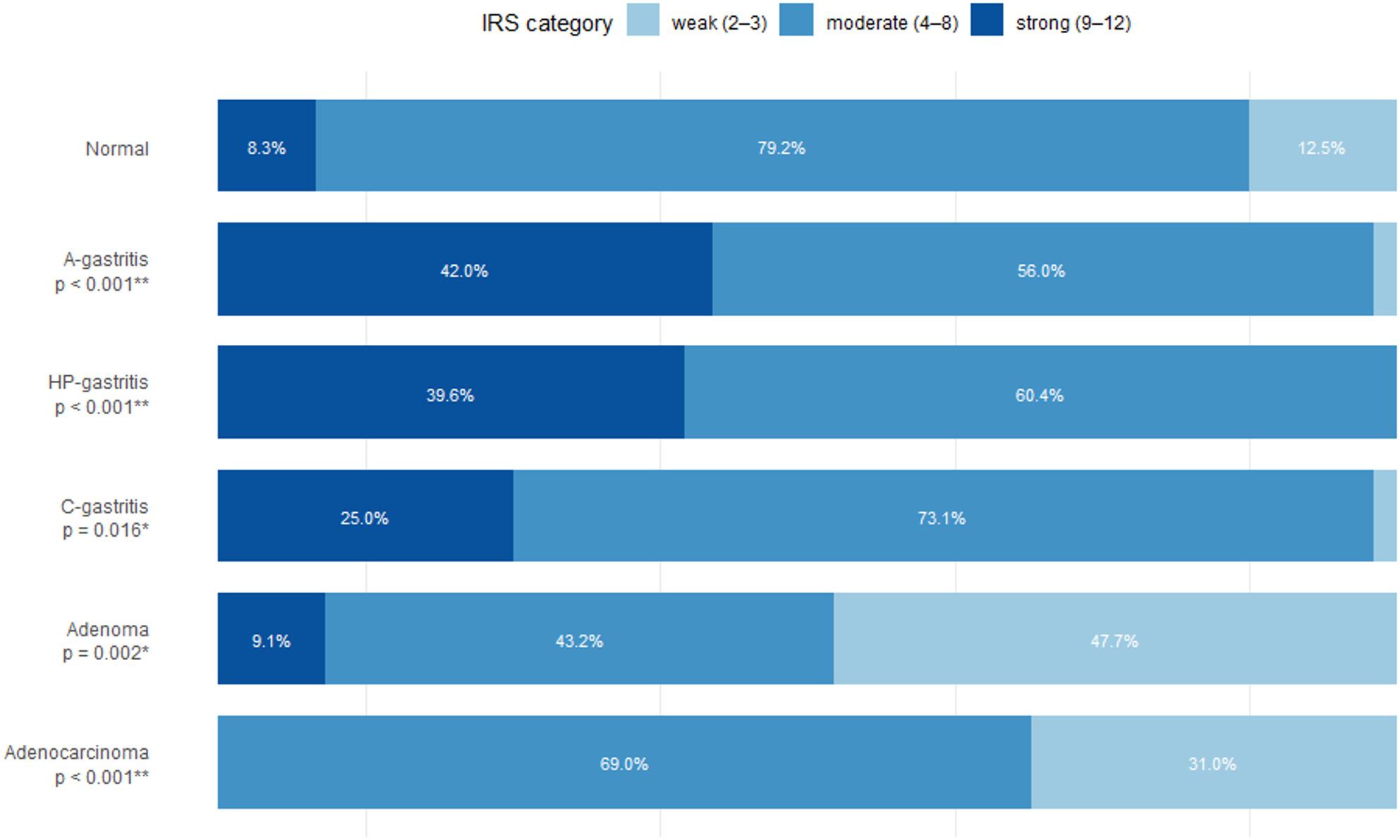
IRS of caspase 7 in different gastric pathologies. Comparison of the percentage of cases across different gastric pathologies (including p-values). Each horizontal bar represents all cases within one entity. Bars are segmented according to staining intensity. Percentages shown inside the bar indicate the fraction of specimens in the corresponding category (values <3% are omitted for legibility)

Nuclear caspase 7 immunoreactivity across diagnostic groups is represented as proportions within the predefined IRS categories (weak, moderate, strong) (Fig. 6). In non-pathological mucosa, staining was predominantly moderate (79,2%) with only few strong cases (8,3%). In inflammatory conditions, A-gastritis showed a marked shift toward higher caspase 7 expression with a large strong fraction (42%), and HP-gastritis displayed a similar increase (39,6%), whereas C-gastritis showed no clear shift toward higher expression (25%). In contrast, neoplastic lesions showed a pronounced reduction of caspase 7, with adenomas dominated by weak to moderate staining (47,7%, 43,2%) and adenocarcinomas showing no strong cases (31%, 69%).

## Discussion

In our cohort, HP-gastritis was characterized by simultaneous upregulation of both USP19 and caspase 7. Chronic active HP-gastritis showed a subepithelial band like infiltration of neutrophilic granulocytes varying from a few granulocytes in between the glands and leucopedesis into the epithelium, and abscess formation combined with a varying degree of chronic inflammatory cells like plasma cells and lymphocytes. In some cases atrophy with and with no basal lymphoid aggregates and intestinal metaplasia can be observed. These criteria are graded according to the updated Sydney system [18]. In line with the concept of chronic active gastritis is the upregulation of USP19 and caspase 7 in our cohort associated with a HP-gastritis. However, activity, atrophy and intestinal metaplasia were not quantitatively graded and tabulated in this study.

Infection-driven gastritis differs from host-related and chemically/reactively induced conditions and points out that these phenotypes follow gastric compartmentalisation (oxyntic corpus/fundus vs. mucosecreting antrum). A-gastritis is typically oxyntic-predominant, with corpus-restricted atrophy and intestinal metaplasia and characteristic enterochromafin-like (ECL)-cell hyperplasia. In our A-gastritis cases, caspase 7 was increased, whereas USP19 remained comparable to normal mucosa. In contrast, chemical/reactive injury may present as ‘reactive gastropathy’, that is, mucosal abnormalities with negligible inflammation, often with foveolar hyperplasia and smooth muscle bundles in the lamina propria [19]. This fits with our C-gastritis findings of reduced USP19 and only a modest, non-significant increase in caspase 7.

In particular, various research approaches suggest that DUB USP19 plays a role in controlling tumor development and the spread of cancer [10]. Here, various mechanisms of cellular USP19 activity have been described. In breast cancer, USP19 promotes migration and invasion by stabilization of the Wnt co-receptor Low-density lipoprotein receptor-related protein 6 (LRP6) and is associated with poor survival [20]. In hepatocellular carcinoma, USP19 overexpression stabilizes the Hippo pathway effector Yes-associated protein 1 (YAP1) which promotes tumor growth and migration and correlates with poor prognosis [21]. Further, in colorectal cancer, USP19 was shown to promote tumor growth by enhancing lipogenesis via the USP19-Malatdehydrogenase 1 (ME1) axis [22]. In another study, USP19 was reported to stabilize Programmed Death-Ligand 1 (PD-L1), thereby reducing T-cell-mediated antitumor immunity [23].

In gastric cancer USP19 overexpression was linked to Matrix-Metalloproteinases 2 and 9 (MMP2/MMP9) up-regulation and tumorigenesis [7]. Our study extends the work by Dong et al. [7] by including in the analysis early stages (A-, C- and HP-gastritis) along the full Correa cascade to show that USP19 activation already occurs in chronic gastritis and adenomas. In addition, we observed in our Central-European cohort both cytoplasmic and nuclear expression, pointing to broader functional roles of USP19 in gastric pathology. By including in our analysis caspase 7 expression we identified low expression of the apoptotic marker caspase 7 reflecting a shift to pro-survival signaling during disease progression.

Outside epithelial tumors, in diffuse large B-cell lymphoma, USP19 has been shown to promote tumor growth by stabilizing the oncogenic protein Parkinson Protein 7 (PARK7). USP19 depletion reduced proliferation, anchorage-independent growth and xenograft tumor formation, whereas USP19 overexpression enhanced these malignant properties [24].

Overall, we provide data showing that overexpression of USP19 correlates with the progression of gastric pathology, culminating most strongly in gastric adenocarcinoma. Furthermore, in our cohort, high USP19 expression was diametrically associated with reduced caspase 7 expression in patients with adenomas and adenocarcinomas. Although we did not examine additional apoptosis (e.g., caspase 3) or proliferation markers (e.g., Ki-67), our data could be of diagnostic relevance for advanced stages of cancer.

## Conclusion

HP-gastritis was characterized by simultaneous upregulation of both USP19 and caspase 7. This pattern likely reflects a regenerative and inflammatory state, which coexists with apoptotic activation. In contrast, adenomas and adenocarcinomas displayed inverse expression of USP19 and caspase 7. From a diagnostic perspective, the high expression of USP19 and significantly reduced expression of caspase 7 indicate inflammatory states and anti-apoptotic signaling in early and late neoplastic transformation.

## Supplementary Information

Supplementary Material 1.

Supplementary Material 2.

## Data Availability

The datasets used and/or analysed during the current study are available from the corresponding author on reasonable request.
