# Supplementary material for: Inverse expression of ubiquitin-specific peptidase 19 and caspase 7 correlates with gastric neoplastic transformation
Source: Diagn Pathol. 2026 Mar 11;21:35. doi: 10.1186/s13000-026-01777-9 (PMC13064426; doi:10.1186/s13000-026-01777-9)
Supplement: Supplementary file 1 — Supplementary Material 1. [file 13000_2026_1777_MOESM1_ESM.docx]

**Supplementary file**

| **Group** | *n* | **Stage 0** | **Stage I** | **Stage II** | **Stage III** | **Stage IV** |
| --- | --- | --- | --- | --- | --- | --- |
| A-gastritis | 52 |  |  | 52 (100%) |  |  |
| H. pylori-gastritis | 53 | 51 (96.2%) | 2 (3.8%) |  |  |  |

**Table S1**: OLGA stage distribution by gastritis etiology.

| **Group** | *n* | **Stage 0** | **Stage I** | **Stage II** | **Stage III** | **Stage IV** |
| --- | --- | --- | --- | --- | --- | --- |
| A-gastritis | 52 | 10 (19.2%) | 9 (17.3%) | 32 (61.6%) |  | 1 (1.9%) |
| H. pylori-gastritis | 53 | 47 (88.7%) |  | 1 (1.9%) | 5 (9.4%) |  |

**Table S2**: OLGIM stage distribution by gastritis etiology.

The table S3 lists, for each diagnostic group, the sample size in the normal reference group and in the respective disease group, the two‑sample Hodges-Lehmann location shift (normal minus disease), its 95 % confidence interval obtained by nonparametric bootstrap resampling, and the Benjamini-Hochberg-adjusted p‑value from the Wilcoxon rank‑sum test. Negative Hodges-Lehmann values indicate higher IRS values in the disease group compared with normal mucosa, whereas positive values indicate higher IRS in normal tissue.

| **n** | | | **n BH-adjusted** | | | |
| --- | --- | --- | --- | --- | --- | --- |
| **Marker** | **Group** | **Normal** | **Group** | **HL** | **95% CI** | **p-value** |
| **cytoplasmic USP19** | A-gastritis | 52 | 52 | 0 | [0, 0] | p=0.364 |
|  | HP-gastritis | 52 | 53 | -1 | [-4, 0] | p=0.001 * |
|  | C-gastritis | 52 | 52 | 0 | [0, 2] | p=0.068 |
|  | Adenoma | 52 | 44 | -4 | [-4, 0] | p*<* 0*.*001 ** |
|  | Adenocarcinoma | 52 | 43 | -2 | [-4, 0] | p*<* 0*.*001 ** |
| **nuclear caspase 7** | A-gastritis | 48 | 50 | -2 | [-4, -2] | p*<* 0*.*001 ** |
|  | HP-gastritis | 48 | 53 | -2 | [-4, -2] | p*<* 0*.*001 ** |
|  | C-gastritis | 48 | 52 | -2 | [-4, 0] | p=0.016 * |
|  | Adenoma | 48 | 44 | 2 | [1, 3] | p=0.002 * |
|  | Adenocarcinoma | 48 | 42 | 2 | [2, 3] | p*<* 0*.*001 ** |

Negative HL indicates higher IRS in the disease group.

**Table S3**: Pairwise comparisons versus normal gastric mucosa for cytoplasmic USP19 and nuclear caspase 7.
